# A Real-Time Comparison of Four Particulate Matter Size Fractions in the Personal Breathing Zone of Paris Subway Workers: A Six-Week Prospective Study

**DOI:** 10.3390/su14105999

**Published:** 2022-05-15

**Authors:** Rémy Pétremand, Guillaume Suárez, Sophie Besançon, J. Hugo Dil, Irina Guseva Canu

**Affiliations:** 1 Department of Occupational and Environmental Health, Center of Primary Care and Public Health (Unisanté), University of Lausanne, Epalinges, 1066 Lausanne, Switzerland; remy.petremand@chuv.ch (R.P.); guillaume.suarez@unisante.ch (G.S.); 2 Régie Automne de Transport Parisien (RATP), 75012 Paris, France; sophie.besancon@ratp.fr; 3 Institute of Physics, Ecole Polytechnique Fédérale de Lausanne (EPFL), 1015 Lausanne, Switzerland; hugo.dil@epfl.ch

**Keywords:** occupational exposure, Bayesian spline model, time-series, public transport, particulate matter, inhalation

## Abstract

We developed a Bayesian spline model for real-time mass concentrations of particulate matter (PM10, PM2.5, PM1, and PM0.3) measured simultaneously in the personal breathing zone of Parisian subway workers. The measurements were performed by GRIMM, a gravimetric method, and DiSCmini during the workers’ work shifts over two consecutive weeks. The measured PM concentrations were analyzed with respect to the working environment, the underground station, and any specific events that occurred during the work shift. Overall, PM0.3 concentrations were more than an order of magnitude lower compared to the other PM concentrations and showed the highest temporal variation. The PM2.5 levels raised the highest exposure concern: 15 stations out of 37 had higher mass concentrations compared to the reference. Station PM levels were not correlated with the annual number of passengers entering the station, the year of station opening or renovation, or the number of platforms and tracks. The correlation with the number of station entrances was consistently negative for all PM sizes, whereas the number of correspondence concourses was negatively correlated with PM0.3 and PM10 levels and positively correlated with PM1 and PM2.5 levels. The highest PM10 exposure was observed for the station platform, followed by the subway cabin and train, while ticket counters had the highest PM0.3, PM1, and PM2.5 mass concentrations. We further found that compared to gravimetric and DiSCmini measurements, GRIMM results showed some discrepancies, with an underestimation of exposure levels. Therefore, we suggest using GRIMM, calibrated by gravimetric methods, for PM sizes above 1μm, and DiSCmini for sizes below 700 nm.

## 1. Introduction

Particulate matter (PM) is a common proxy indicator for air pollution. It consists of a complex mixture of solid and liquid particles of organic and inorganic substances suspended in the air. PM affects more people than any other pollutant [1]. Short-term exposures to coarse (PM10, i.e., particles with an average aerodynamic diameter < 10 µm) and fine (PM2.5, i.e., <2.5 µm) particles are clearly associated with all causes of cardiovascular, respiratory, and cerebrovascular mortality [2], while long-term PM exposure increases the risk of mortality from cardiovascular disease, respiratory disease, and lung cancer [3]. These associations persist below the exposure level outlined in the 2006 WHO guideline [4]. This led the WHO to reduce the recommended maximum annual average exposure level for PM2.5 from 10 µg/m^3^ to 5 µg/m^3^ and for PM10 from 20 µg/m^3^ to 15 µg/m^3^ [5]. The recommended maximum 24-hour average exposure was reduced from 25 µg/m^3^ to 15 µg/m^3^ for PM2.5 and from 50 µg/m^3^ to 45 µg/m^3^ for PM10 [5]. Regarding levels of smaller particles (PM1 and PM0.1, i.e., <1 µm and 100 nm, respectively) that are beyond the guideline exposure levels, increasing epidemiological evidence suggests an association between short-term exposures and negative impacts on cardiorespiratory health, as well as the health of the central nervous system [6]. 

Epidemiological and toxicological studies show varying types and degrees of health effects related to PM, suggesting a role for both the chemical composition (such as transition metals and combustion-derived primary and secondary organic particles) and physical properties (size, shape, and surface area) along with concentration [7,8,9,10,11,12,13,14,15,16,17]. Yet, the research in this field is limited and controversial, particularly when comparing the results from epidemiological and experimental (in vivo and in vitro) studies [18]. This is especially true for PM in underground subway systems, where PM concentrations can be significantly higher than outdoors and have a very specific physio-chemical composition and size distribution [19,20,21]. Ultrafine particles (UFP) are the strongest contributor to subway pollution when the particle number concentration is used as the exposure metric [22]. Because of this size distribution and a highly ferruginous composition, along with the presence of trace metals (Mg, Al, Si, Ti, V, Cr, Mn, Ni, Cu, Zn, Ba, and Pb) [23,24], subway PM generates more reactive oxygen species (ROS) and oxidative-stress-related outcomes compared to other PM [18,25]. A comprehensive assessment of individual exposure to subway PM, particularly the finest size fractions, is urgently warranted in order to identify the sources and factors that contribute to high PM levels in individual subway stations and lines [26,27]. While the potential health impacts of subway PM on workers and/or commuters remain uncertain, exposure assessment studies are essential for risk assessments and exposure control interventions.

A Franco-Swiss epidemiological research project called “ROBoCoP” (Respiratory disease Occupational Biomonitoring Collaborative Project) was launched at the Parisian urban transport company (RATP) to address this issue [28]. Within this project, a 6-week longitudinal study was conducted among RATP workers to measure their personal exposures in terms of particle number and particle mass concentration using direct-reading instruments along with standardized gravimetric analysis [29]. The application of a Bayesian spline method to the collected UFP number measurements and contextual data enabled estimations of the differences in UFP exposure between subway professionals, stations, and various locations [22]. The developed model proved informative for documenting the magnitude and variability of UFP exposure and for understanding exposure determinants. 

In the present study, we build on this existing work and aim to show that it can also be applied to other size fractions and measurement techniques, thereby demonstrating the general applicability of the model. Therefore, we apply a Bayesian spline method to the real-time mass concentrations of PM10, PM2.5, PM1, and PM0.3 measured simultaneously by an optical particle counter, and we analyze the exposure profiles and determinants of these aerosol fractions in the personal air samples of Parisian subway workers. In addition to covering the acute problem of PM exposure, our study also has a more general perspective. It gives insight into the limitations and intercomparability of different measurement techniques and, as such, can help design future studies.

## 2. Materials and Methods

### 2.1. Data Collection

Data were collected in the frame of a longitudinal pilot study dedicated to a comprehensive exposure assessment, as described in the study protocol [28]. We focused primarily on subway line 7. Line 7 entered into operation in 1910 and crosses Paris from the northeast to the southeast following a slightly curved route. This entirely underground line is one of the longest (22 km and 38 stations) and busiest (more than 136 million yearly passengers) in the Parisian subway network.

Nine subway professionals, who primarily work on line 7, were included from three different occupations: station agents (n = 3), locomotive operators (n = 3), and security guards (n = 3). Their tasks and exposure results were described in detail elsewhere [28,29]. The data collection lasted for a total duration of 6 weeks (from 7 October to 19 November 2019, i.e., 2 weeks per type of subway professional). RATP safety regulations do not allow any RATP professionals to wear any equipment other than what is used for their regular work. Therefore, airborne PM were collected as close as possible to the worker’s personal breathing zone (PBZ) with appropriate equipment carried by two or three RATP technicians who job-shadowed RATP workers for their entire 6–8-hour shifts. 

For the continuous measurement of airborne particles, we used the portable GRIMM Aerosol Spectrometer and Dust Monitor (GRIMM Aerosol Technik, Ainring, Germany) Model 1.109, which is considered a research-grade device of moderate cost [30,31,32]. The measuring principle of this model is based on light scattering off single particles with a semiconductor laser as a light source. Model 1.109 possesses 31 size channels for measuring particle size distribution within the range of 0.25 to 20 µm, recorded every 5 min (with a time resolution of 6 s). For each size fraction, the mass concentration is estimated. For these reasons, GRIMM Model 1.109 is considered suitable for aerosol research and the compilation of occupational health data [30,31,32]. As GRIMM results are not in compliance with European standards for PM10 and PM2.5, we complemented their measurement by standard gravimetric analysis (EN 12341). For this, the sampling train was equipped with a filter (PTFE Membrane Filters (37 mm), Sigma-Aldrich, Molsheim, France) in a cassette holder (Personal Impactor H-PEM, BGI, USA) connected to a cyclone and attached with flexible tubing to a pump (GilAir Plus, Sensidyne, Germany) operating at 4 L/min. Moreover, we used the particle counter “DiSCmini” (Testo, Monchaltorf, Suisse) to measure particles from 10 to 700 nm, yielding particle number concentration (#/cm^3^) and lung-deposited specific area (LDSA) (recorded every 6 s; time resolution of 1 s). In addition to instrumental PM measurements, technicians documented every participant’s location and event that occurred during his/her work shift in a standardized activity logbook. 

### 2.2. Data Management

The PM records and activity logbooks were processed as follows. First, we defined the time-series from daily collected PM measurements, and each time-series corresponded to a complete 6-hour work shift, linked with an activity logbook. The calibration of GRIMM measurements with reference to PM10 or PM2.5 levels is recommended, although not yet standardized [33,34]. In this study, we applied the most recent method [26] to standardize the PM10 or PM2.5 time-series using the temperature and relative humidity measurements as well as the gravimetric concentrations of PM10 or PM2.5. We tested several regression functions and determined that the power function had the highest $R^{2}$ fit to both the PM2.5 and PM10 gravimetric concentrations (Supplementary Figure S1). However, given the absence of measurement standards for the calibration of PM1 and PM0.3, we also used non-calibrated, raw time-series data to compare the aerosol dynamics according to the size fraction. To analyze their variation, three independent variables were extracted from activity logbooks: *Station*, *Environment*, and *Event*, along with their corresponding timing and duration. The *Event* variable documented the events that occurred during the recording (e.g., exposure to tobacco smoke, intervention on ticket distributor, subway cabin heater, train passing), as previously described [22]. The *Environment* variable defines the type of locality, or setting, that the participant was located in or visited during his/her work shift (e.g., sampling room, cloakroom, ticket-counter, underground corridor, subway platform). The variable *Station* corresponds to the participant’s location in the subway rail network. When traveling underground between two subway stations on the same line, the *Station* variable was set to Tunnel for all corresponding time points.

In order to better understand the PM variations between subway stations, additional variables were retrieved from RATP records, namely the year of station opening, the year of the last station renovation, the annual number of passengers entering the station in 2019, and the number of ventilators per station, as well as the factors contributing to the natural station ventilation, such as station topography, the number of entrances, the number of correspondence concourses, the number of platforms and tracks, and the type of station design. The latter was assessed according to [35] and completed to account for local architectural particularities, with seven types of station design (coded from A to G) overall.

### 2.3. Statistical Analysis

To explore the association between PM mass concentration and independent variables, we developed a Bayesian spline model, as previously described [22]. We fitted four separate models that considered the log10 transformed PM10, PM2.5, PM1, and PM0.3 time-series as dependent variables and *Station*, *Environment*, and *Event* as independent variables, with an inter-day-specific intercept absorbing the corresponding job random effect:

(1) $$\begin{array}{c} Y_{ir}\;\sim \;N\left\{\mu_{i}+XStation_{ir}^{T}\alpha +XEnvironement_{ir}^{T}\beta +XEvent_{ir}^{T}\gamma +\zeta_{i}^{T}b\left(t_{\mathrm{ir}}\right),{\;\sigma }^{2}\left(Day_{i}\right)\right\}. \end{array}$$

The models were fitted within a Bayesian framework and strictly validated using the “When to worry and how to Avoid the Misuse of Bayesian Statistics” (WAMBS) checklist [36]. This validation consisted of a convergence check for all 117 parameters (i.e., 45 stations, 8 locations, 10 events, 24 inter-day-specific intercepts, 24 inter-day-specific variances, and the 3 intercept and 3 variance parameters of the job random effect) using Gelman and Rubin convergence diagnostics and by visualizing the trace and density plots of all coefficients except the many ζ coefficients. A sensitivity analysis was performed on the prior distribution of α, β, and γ coefficients by varying the standard deviation from 5 to 3 or 10. In addition, we checked for large degrees of autocorrelation in the Markov chain using autocorrelation plots with lags varying from 1 to 20. Finally, we conducted a posterior predictive checking step by predicting the particle number concentration for complete time-series using the input data and then comparing it with the observed particle number concentrations. 

Moreover, we explored the station-related variables described above as explanatory factors of β coefficients obtained by modeling. For this, we assessed the Pearson correlation coefficients. Given the exploratory and not hypothesis-driven analytical framework adopted here, we applied no correction for multiple comparisons.

All data management and statistical analyses were performed using the R software package (version 3.6.2). In the Bayesian inference step, we used the R2Jags library and JAGS standard software with the model described in Bayesian inference Using Gibbs Sampling (BUGS) format (10.16909/DATASET/28, File S1).

## 3. Results

### 3.1. Descriptive Results

**Figure 1:**
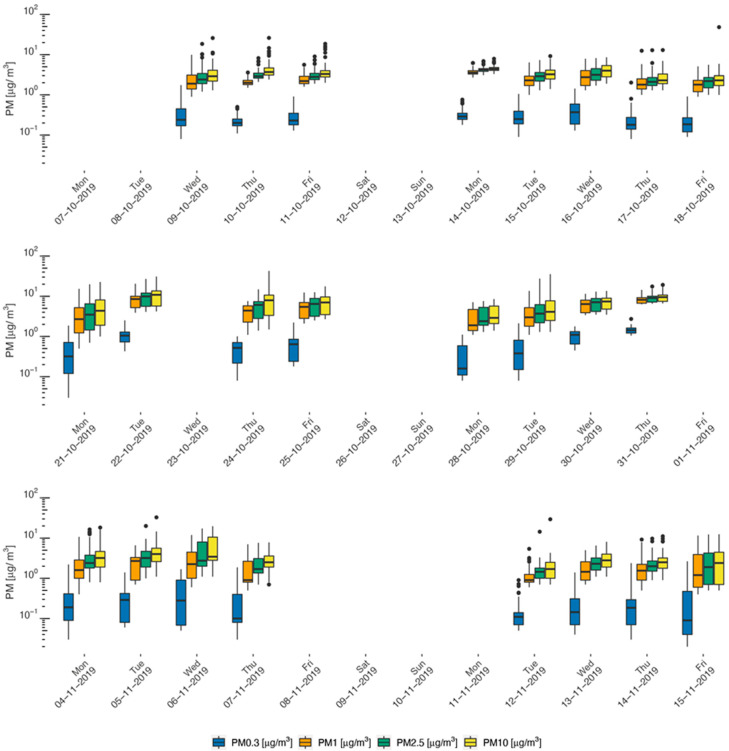
PM daily concentrations measured by GRIMM in the personal PBZs of Parisian subway workers: station agents (**top**), locomotive operators (**middle**), and security guards (**bottom**).

Figure 1 summarizes daily PM mass concentrations for the four size fractions measured by GRIMM over six weeks in the PBZ of subway workers, stratified by their job. The values of the daily PM mass concentration per size fraction can be consulted at the Unisanté Research Data Repository (10.16909/DATASET/28, Excel File 1). It is worth noting that no real-time measurement records were available for two days out of ten for every job. This was due either to the difficulties in GRIMM use in the first two days of the field campaign (despite the fact that all RATP technicians were trained and well-experienced), uncontrolled disruptions in the dust monitor’s functioning, or an unexplained stoppage of measurement recording. According to the available records, station agents had the most precise estimates of their daily individual exposure (appearing with the narrowest confidence intervals around the central estimates in Figure 1), regardless of the PM size fraction. This was in line with our previous study and was due to the relative stability of their tasks and the fixed nature of their workplace (in the ticket counter or its surrounding environment) [22]. As expected, the PM0.3 daily concentration was more than an order of magnitude lower compared to the other PM fractions, and this difference was consistent over time and across jobs. This is explained by the fact that the ultrafine particle contribution to the PM mass is very limited [37]. The variability in the daily mass concentration was the highest for PM0.3, followed by PM1. For PM2.5 daily mass concentration, the variability was the lowest overall; however, this was not consistent, as for some days PM10 mass concentration showed less variability than PM2.5. Overall, PM10 exposure, estimated as mass concentration, was predominant over that of the other PM size fractions in all jobs, closely followed by PM2.5 (Figure 1). 

The correlations between gravimetric and real-time PM2.5 and PM10 measurements were rather fair (Supplementary Material, Figure S1), and this result is in line with previous studies of calibration issues [34,38,39,40]. When comparing the PM2.5 and PM10 concentrations before and after calibration (10.16909/DATASET/28, Excel Files 2 and 3, respectively), the geometric means estimated based on raw real-time records appeared unrealistically low (Excel File 3, sheet “GM_GSD_day”). Moreover, even after calibration, the real-time measurement of PM2.5 and PM10 underestimated the personal exposure in locomotive operators and security guards when measured as gravimetry mass [29], and this underestimation seems more important for PM10 levels. For instance, the geometric mean for PM10 of locomotive operators was 4.24 μg/m^3^ before calibration, 88.12 μg/m^3^ after calibration (10.16909/DATASET/28, Excel Files 2 and 3, sheet “GM_GSD_job”), and 188.50 μg/m^3^ when measured by the gravimetric method [29].

**Figure 2:**
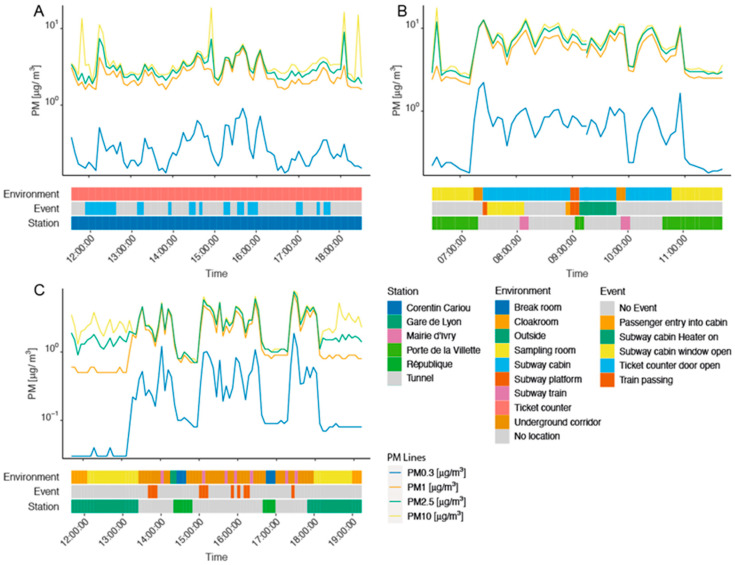
Real-time measurements of PM mass concentrations over the work shift in the PBZ for station agents (**A**) (11 October 2019), locomotive operators (**B**) (25 October 2019), and security guards (**C**) (7 November 2019).

Figure 2 illustrates the integration of the contextual information collected through the daily activity logbooks to explain the variation in personal PM concentrations over the workers’ work shifts. For the sake of clarity and comparability, we plotted data collected during the last day of the first week of exposure monitoring in each job, corresponding to the middle of the monitoring period. Professionals’ work shifts usually started and ended in the sampling room at “Porte de la Villette” (Figure 2B,C), where the PM mass concentration is very low compared to other environments. On the 11 October 2019, all station agent PM records corresponded to the PM mass concentration in the ticket counter situated at the station Corentin Carriou (Figure 2A). The only recorded event this day was the opening of the ticket counter door, which consistently increased the PM mass concentration of all PM sizes fractions, particularly for PM10. It is remarkable that all PM size fraction mass concentrations evolved almost in parallel, although the increases in PM0.3 observed in locomotive operators and security guards (Figure 2B,C) were greater than those of other PM. 

The first PM0.3 peak in Figure 2B is particularly large, corresponding to the walk from the sampling room via the underground corridor before reaching the train and entering the cabin. Opening the window during the initial phase of driving the train seemed to decrease the PM mass concentration of all size fractions, while putting the heater on increased all types of PM in a very similar way. For the security guard, PM concentrations changed with environment, and every passing train event appeared to be followed by a peak in all PM size fractions, particularly for PM0.3 (Figure 2C). The shape of PM variation in this illustrative time-series clearly requires a model supporting the non-stationarity autocorrelation while taking into account different fixed effect variables (*Station*, *Environment*, and *Event*), and it confirms the relevance of the Bayesian spline model.

### 3.2. Bayesian Modeling Results

The fitting of the Bayesian spline model to the personal PM time-series resulted in a good mixing behavior in Markov chains. The model validity was supported by low Gelman and Rubin convergence diagnostics and autocorrelations, conducted in accordance with the WAMBS checklist [36]. Based on the visual examination of the trace and density plots of coefficients, we identified no conditions invalidating our models. Furthermore, we found that modifying the prior distribution for different parameters did not impact the estimation of the posterior distribution, thus demonstrating the robustness of the model. All of the estimated parameters of this model are available from the Unisanté Research Data Repository (10.16909/DATASET/28, Excel File 3, sheets “alpha_station”, “beta_environments”, and “gamma_event”). The model coefficients obtained when fitting the model to calibrated data were very similar (10.16909/DATASET/28, Excel File 2). 

**Figure 3:**
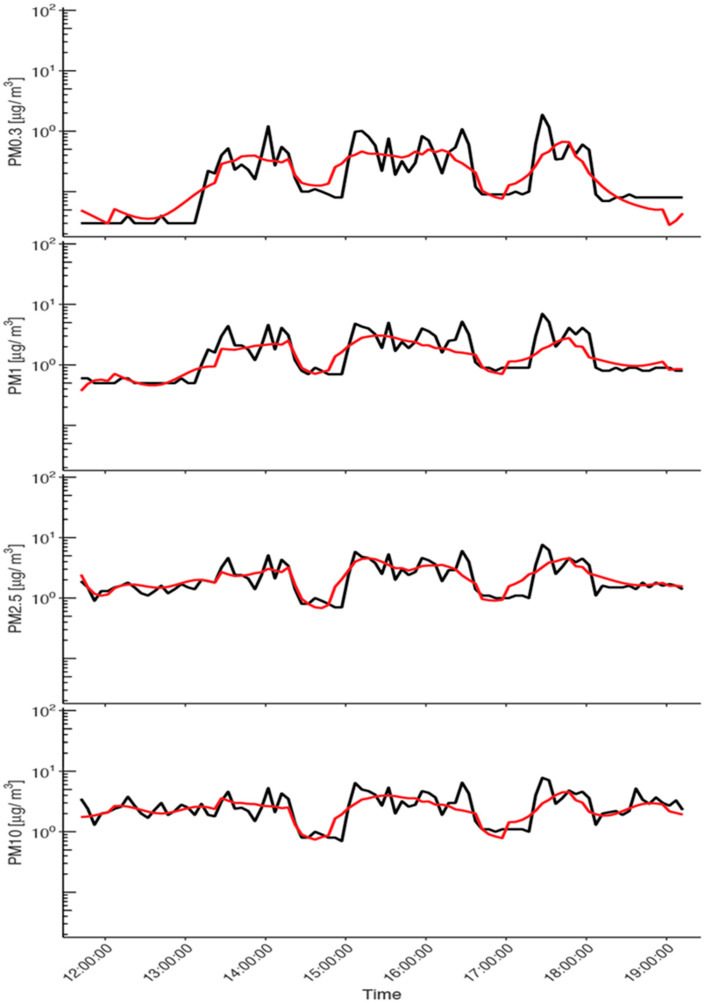
Prediction of the Bayesian spline model. Visualization of the Bayesian spline model prediction (red curve) for the personal PM10, PM2.5, PM1, and PM0.3 mass concentrations of the security guards on the 7 November 2019.

Figure 3, using the last example of the security guards’ exposure monitored on the 7 November 2021, shows that the prediction of the PM mass concentration by the Bayesian spline model overlaps reasonably well with the observed values for all size fractions. Visually, the fit accuracy looks similar across size fractions, where some of the highest observed concentration peaks are above the model prediction curve. This is particularly the case for PM0.3. Although satisfactory according to the WAMBS guidelines [36], this model fit is worse than the fit obtained in our previous study on PM0.3 number concentration measured by DiSCmini [22]. This is due to the fact that fewer data were recorded by GRIMM, and thus available for model training, because of a lower time resolution of measurement recording compared to DiSCmini (5 min versus 6 s) and six days with missing GRIMM records. 

**Figure 4:**
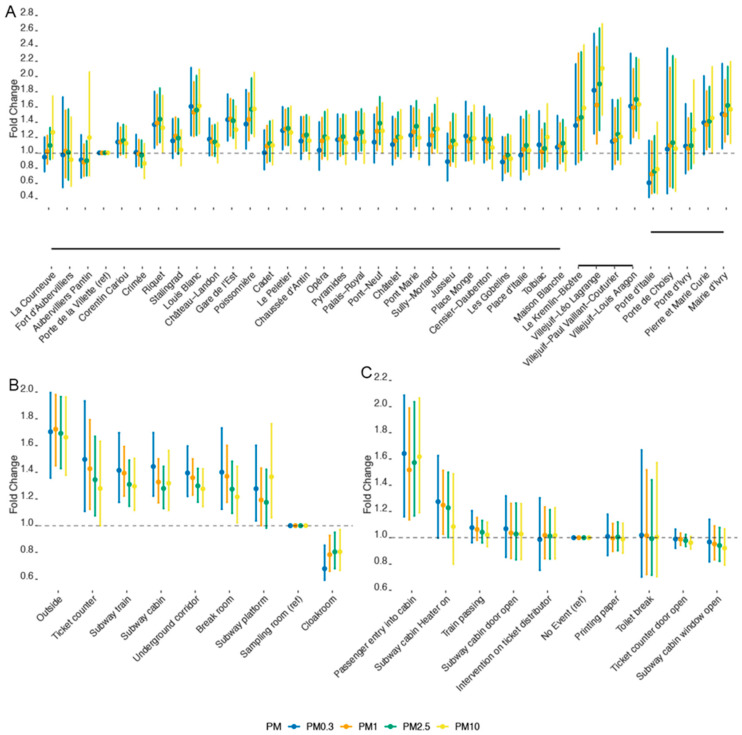
PM mass concentration variation on subway line 7 for *Stations* (**A**), *Environments* (**B**), and *Events* (**C**). The plots represent the posterior distribution of the coefficient transformed as fold change (10^coefficient^) for every category of the studied factors. The bar is the 95% credible interval, and the point is the median of that distribution. The bottom sub-panel in panel (**A**) represents the subway line 7. The discontinuity shown at the “*Maison Blanche*” station corresponds to two embranchments, one towards “*Villejuif-Louis Aragon*” and the second towards “*Mairie d’Ivry*”. The reference category is noted with (ref).

Figure 4, panel A represents the posterior distribution of the estimated coefficients for every subway station along line 7. The coefficients are expressed as a fold change (10^coefficient^) with respect to the reference station, Porte de la Villette, for each size fraction. The personal mass concentrations measured at this station were 0.31 ± 2.86 μg/m^3^ for PM0.3, 2.71 ± 2.00 μg/m^3^ for PM1, 3.32 ± 2.00 μg/m^3^ for PM2.5, and 3.91 ± 2.03 μg/m^3^ for PM10. The calibrated values for PM2.5 and PM10 were 70.08 ± 1.52 μg/m^3^ and 82.66 ± 1.85 μg/m^3^, respectively. 

In line with the results of descriptive analysis, the coefficients corresponding to the different PM size fractions are rather similar at most subway stations (Figure 4A). However, a closer look reveals that PM2.5 raises the highest exposure concern, with 15 stations out of 37 showing significantly increased mass concentrations compared with the reference station. The coefficients corresponding to these PM2.5-polluted stations have credible intervals above 1. Twelve of these stations also had significantly higher PM1 mass concentrations compared to the reference. Regarding PM10, only 7 stations out of 37 had significantly higher mass concentrations, and an additional 4 stations presented an increase with borderline credibility. Finally, with respect to PM0.3, eight stations were significantly more polluted than the reference station. It is noteworthy that the magnitude of change in PM mass concentration between stations was not high and was rarely greater than 10% (Figure 4A). In this respect, the most polluted stations were Villejuif-Léo Lagrange, with the highest levels of PM10, PM2.5, and PM0.3; Louis Blanc, with the second-highest mass concentrations of PM0.3 and PM10; and Villejuif-Louis Aragon, with the highest PM1 and the second-highest PM2.5 and PM0.3 mass concentrations. Further analysis of the station characteristics revealed no correlation with the annual number of passengers entering the station, the year of station opening or renovation, or the number of platforms and tracks. In contrast, we observed a consistent negative correlation with the number of entrances for all size fractions (−0.11 for PM0.3, −0.15 for PM1, −0.24 for PM2.5, and −0.06 for PM10). The number of correspondence concourses for other stations was negatively correlated with PM0.3 and PM10 (−0.07 and −0.03, respectively) and positively correlated with PM1 and PM2.5 (0.34 and 0.06, respectively). Station types C and F were associated with the highest levels of PM in all size fractions, whereas high PM0.3 concentrations were also observed in type A stations and PM10 in type G stations (results not shown). These findings are in line with the previously reported data suggesting the importance of general (natural) ventilation, which can be determined by the station architecture and topography [35]. Regarding the latter, the correlation coefficients between PM coefficients and minimal station altitude decrease with increasing PM size (0.28 for PM0.3, 0.19 for PM1, 0.06 for PM2.5, and 0.08 for PM10). 

Figure 4B shows that, with the exception of the cloakroom, all studied environments had higher PM exposure compared to the sampling room, although the highest levels of PM were measured outdoors. The highest PM10 exposure was observed at the station platform, followed by the subway cabin and train, while ticket counters had the highest PM0.3, PM1, and PM2.5 mass concentrations. Regarding the effect of studied events on the PM level (Figure 4C), only the event called “Passenger entry into cabin” was associated with a significant increase in the mass concentration of all size fractions. Indeed, despite the name, this event corresponds to opening the train cabin door to enter the cabin or have a study technician bring in some equipment; passengers, who should not disturb the locomotive operator, did not enter. Turning the heater on was also associated with a concentration increase, but only for fine and ultrafine particles. The increase in PM mass concentration when or after a train was passing or a cabin door was opened was small and not statistically significant.

### 3.3. Comparison of GRIMM and DiSCmini Results

It is noteworthy that in our previous study of UFP number concentration, we observed the opposite effect for the train passage, which lowered the UFP number concentration measured with DiSCmini [22]. The other increases in particle number concentration were identified in the same environments as in this study, although with different absolute concentrations. For instance, the subway platform was found to be more polluted with UFP than the ticket counter, identified as the second most UFP-polluted environment in that study, while the outdoor UFP number was not significantly higher than the reference UFP concentration [22]. In order to investigate these discrepancies, we compared GRIMM and DiSCmini measurement results in terms of particle number concentrations. For this, we integrated the GRIMM number concentration values—from 0.25 to 0.70 μm—in the particle size range corresponding to the DiSCmini operating range (0.01 to 0.7 μm) (Supplementary Material, Figure S2, 10.16909/DATASET/28, File 4). 

**Figure 5:**
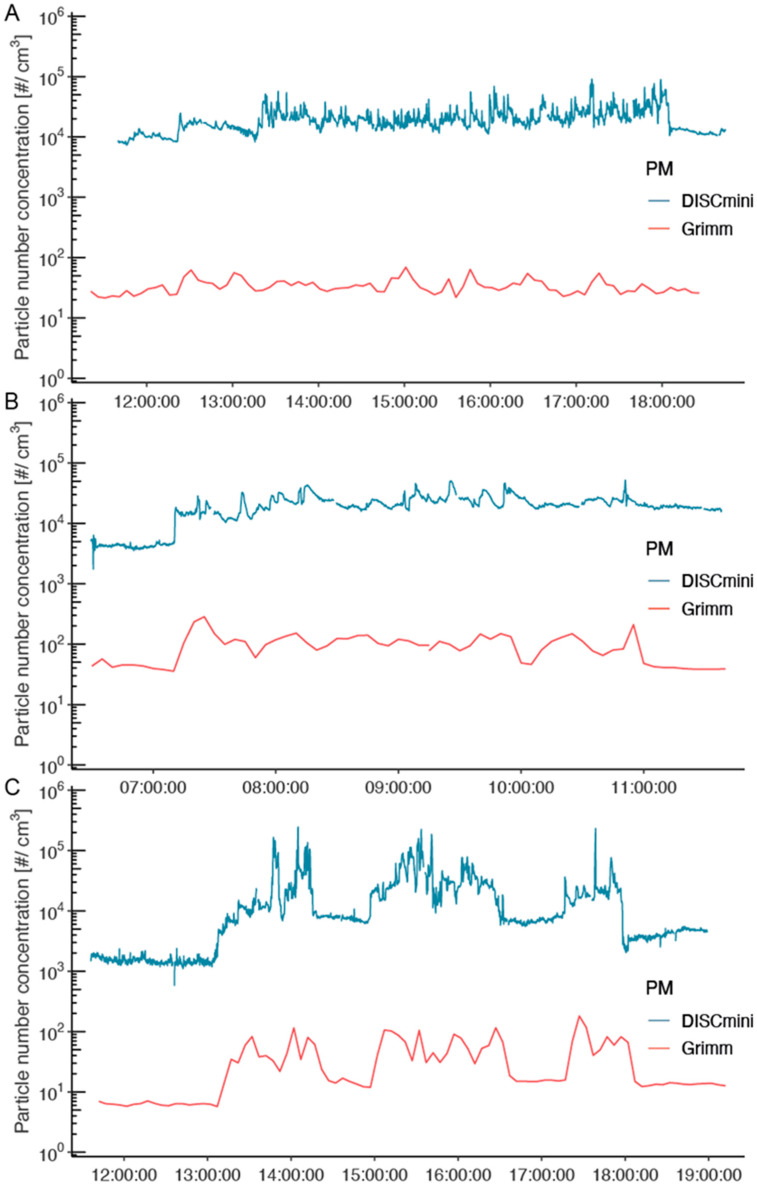
Total particle number concentration measured by DiSCmini for sizes from 0.01 to 0.7 μm (blue) and by GRIMM for sizes from 0.25 to 0.7 μm (red) in the personal breathing zone samples of Parisian subway workers. (**A**) Station agents (10 October 2019), (**B**) locomotive operators (25 October 2019), and (**C**) security guards (7 November 2019).

As illustrated in Figure 5, using the measurements corresponding to the mid-point days in the PM monitoring interval, the two devices reflect relatively similar patterns in concentration changes over time. Although the latter records more peaks, probably due to a better time resolution (lower averaging span), the increases in concentration recorded with GRIMM correspond to increases in DiSCmini measurement results. Quantitatively, the particle number concentrations measured by the DiSCmini are three orders of magnitude higher than the ones measured by the GRIMM. This observation can be explained by the size distribution of the analyzed environmental aerosols, where the contribution of ultrafine particles (<100 nm) to the overall number concentration is particularly high [18,21,24]. Burkart et al. showed that, due to the number size distribution of urban environment aerosols, GRIMM (model 1.109) only measures a tiny part (6%) of the total particle number determined via a Vienna-type differential mobility analyzer (DMA) [41]. Although the particle number concentrations obtained with DiSCmini and GRIMM follow the same trends, particles with aerodynamic diameters below the GRIMM lower cut-off (0.25 µm) are expected to contribute largely to the aerosol distribution, thereby explaining the differences in Figure 5.

## 4. Discussion

### 4.1. Detection Physical Principles

Most of the commercially available direct-reading particle counters rely on a few physical principles that determine their strengths and limitations in a given context of use [39]. In a very generic manner, the use of instruments based on different physical principles implies questions to consider beyond the manual instructions: (i) which physical event generates a measurable change, and (ii) what is the nature of this change? In the case of particle counters, OPCs such as the GRIMM rely on light scattering properties inherent to particles in the micron-domain, with an exponential decrease in the scattering intensity with the particle diameter. Here, the instrument’s lower cut-off diameter of about 0.25 µm is explained by the Mie diffusion domain at the laser wavelength. The scattering-based approach implies that the size determination is sensitive to a series of physical variables such as refractive index, density, and shape of particles. In addition, the conversion of the particle number distribution into mass concentration is done by applying a mathematical model with approximated values for particle density and morphology. Thus, in addition to the standard calibration (dolomite dust), the GRIMM instrument enables the possibility of adjusting the density variable through the correction-factor (C-factor) adapted to a particular aerosol [34]. Despite this measurement correction approach, one assumption still remains: namely, that the sensitive physical variables—density, refractive index, and shape—are unchanged during the period of measurement. For environmental, non-standardized aerosols, this assumption is even more problematic because a direct link between particle size and chemical composition, and thus refractive index and reflectivity, can be expected. An earlier comparison study performed in urban environments reports that GRIMM underestimates the mass concentration by about 20% when considering gravimetric data as reference and a comparable particle size range [41]. In accordance with this, in the present work, the GRIMM results underestimated PM2.5 and PM10 exposure as compared to the gravimetric method, and this was the case for both calibrated and non-calibrated comparisons. However, the positive effect of calibration on GRIMM measurements was clearly visible as it significantly reduced the gap with corresponding gravimetric data for both PM fractions.

In turn, personal monitors such as DiSCmini, Partector, or NanoTracer rely on the electrical measurement (current intensity) of the particle-borne charges resulting from the initial electrical diffusion charging of the aerosol. Since the electrical diffusion charging behavior of submicron particles is size-dependent, these devices provide quantitative information on both the particle number concentration and the modal diameter in the UFP domain (typically from 20 to 400 nm). The conversion of electrical mobility into aerodynamic diameter requires a series of approximations of the particle shape (spherical) and the size distribution (lognormal). Considering the ultrafine condensation particle counter (UCPC) as a reference instrument, Todea et al. showed that the DiSCmini overestimates the number concentration by about 30% in the aforementioned size range [42]. The same authors identify as interfering variables the presence of particles with sizes > 400 nm—discarded via the use of an impactor—and aggregates in the aerosol for which the charge is greater than for the equivalent non-aggregated particle. Similarly, Mills et al. reported a deviation from reference—here the scanning mobility particle sizer (SMPS)—for DiSCmini number concentration of about 21% in the case of polydisperse aerosols [43]. 

### 4.2. Qualitative Reliability of Measurements and Inter-Device Comparison Issues

It can be argued that the data obtained with the GRIMM and DiSCmini shown in Figure 5 follow a similar trend and that differences are due to the time resolution and different sensitivities to UFP for the two techniques. However, this cannot explain the opposite results for the “train passing” event obtained from the model, nor the quantitatively different response to other events and environments. A closer inspection of Figure 5 reveals some hints about the qualitative differences in the responses of both methods. For example, around 14h00 in Figure 5C, DiSCmini shows a dip where GRIMM shows a peak, similar to around 10h00 in Figure 5B. Such comparisons for both methods, including *Station*, *Location*, and *Event* indicators, can be found in the full online data set (10.16909/DATASET/28, File 4), and some representative mismatches are reproduced in Figure S3. Many occasions can be found where DiSCmini and GRIMM show opposite trends, and the measurements on 30 October 2019 even show a completely different shape. This clearly indicates a problem with the direct comparison of data from both methods.

Based on the qualitative consistency of the data, our opinion is that the results from DiSCmini are more representative of the real submicron PM concentration. This is based on the observation that all DiSCmini measurement sessions show a similar type of behavior with features clearly related to *Event*, *Location*, or *Station* variables, whereas the GRIMM data appear to show a less structured response. This is directly reflected in the Bayesian model fits and the uncertainties in the changes to all model parameters. Furthermore, the GRIMM results show almost identical curves, different by a multiplication factor, for all PM sizes (Figure 2). This suggests that results for the smallest particle range are strongly influenced by the presence of PM10 and PM2.5. These factors lead us to conclude that the GRIMM is not well suited to characterize PM concentrations for sizes below 700 nm in a typical underground aerosol environment with a mixture of particle sizes and compositions. Instead of a straightforward interpretation of the particle number concentration that considers the full operational capability of each instrument, the rational delimitation of the optimal particle size range for both the GRIMM and DiSCmini enables a useful co-deployment covering all the aerosol dimensions. 

## 5. Conclusions

One of the aims of our study was to use the GRIMM as an intermediate link between the standardized gravimetric method for coarse and fine particles and the real-time measurement method for UFP using DiSCmini. Unfortunately, the large discrepancy in trends between GRIMM and DiSCmini for PM with sizes below 700 nm (the upper limit of the latter method) renders this approach unfeasible. Given the high quality of the Bayesian model fit for the DiSCmini, we have good confidence in this method for real-time measurements of UFP. However, an independent calibration would be needed to determine absolute particle concentrations [30,40]. This goes beyond the scope of this study, but one could envision combining DiSCmini measurements with scanning electron microscopy (SEM) or transmission electron microscopy (TEM) analysis on representative samples.

Another goal of this study was to independently measure the PM concentrations of four different size fractions in a real-time fashion as a function of various parameters applicable to workers in the Parisian subway. The GRIMM promises exactly this functionality; however, based on the results discussed above, it is clear that a single device is not suitable for this aim, and further instruments are needed. We suggest using a GRIMM, previously calibrated by gravimetric methods in the environment of interest, for PM sizes above 1 μm, and a DiSCmini for sizes below 700 nm. This co-deployment of DiSCmini and GRIMM in urban sites delivers valuable information on the dynamic evolution of the aerosol in terms of the number concentration and size distribution (or modal size), covering a large size domain from ultrafine to fine particles. Such dynamic information is particularly useful for identifying possible sources of emissions and for understanding the interventions/changes that govern the aerolics of the system.

Despite the above-discussed discrepancies between the measurement results of GRIMM and the gravimetric method on the one hand and GRIMM and DiSCmini on the other hand, we demonstrated the relevance of our Bayesian spline model for analyzing four time-series of PM concentrations according to subway stations, locations, and events. The strengths of this study lie in the assessment of personal (breathing zone) exposure using multiple devices and ad hoc modeling of exposure variations for four PM sizes simultaneously. This enabled us to evidence a differential exposure profile in terms of PM sizes in subway stations and workplaces, a singular behavior of UFP compared with fine and coarse particles. Further effort should be focused on the development and improvement of portable and affordable devices as well as calibration methods that provide reliable estimates for different exposure components in complex environments, such as underground railways.

## Data Availability

All data related to this study are available at 10.16909/DATASET/28.

## Supplementary Materials

The following supporting information can be downloaded at: https://www.mdpi.com/article/10.3390/su14105999/s1, Figure S1: Regression analysis between the simultaneously collected gravimetric and GRIMM data; Figure S2: Particle number concentrations measured in personal samples; Figure S3: Further comparison between DiSCmini and GRIMM.

## References

[1] WHO (2021). Ambient (Outdoor) Air Quality and Health. https://www.who.int/news-room/fact-sheets/detail/ambient-(outdoor)-air-quality-and-health.

[2] Orellano P., Reynoso J., Quaranta N., Bardach A., Ciapponi A. (2020). Short-term exposure to particulate matter (PM10 and PM2.5), nitrogen dioxide (NO_2_), and ozone (O_3_) and all-cause and cause-specific mortality: Systematic review and meta-analysis. Environ. Int..

[3] Chen J., Hoek G. (2020). Long-term exposure to PM and all-cause and cause-specific mortality: A systematic review and meta-analysis. Environ. Int..

[4] WHO (2006). Air Quality Guidelines for Particulate Matter, Ozone, Nitrogen Dioxide and Sulfur Dioxide. Global Update 2005. Summary of Risk Assessment.

[5] WHO (2021). Global Air Quality Guidelines. Particulate Matter (PM2.5 and PM10), Ozone, Nitrogen Dioxide, Sulfur Dioxide and Carbon Monoxide.

[6] Bencsik A., Lestaevel P., Guseva Canu I. (2018). Nano- and neurotoxicology: An emerging discipline. Prog. Neurobiol..

[7] Guo C., Lv S., Liu Y., Li Y. (2022). Biomarkers for the adverse effects on respiratory system health associated with atmospheric particulate matter exposure. J. Hazard. Mater..

[8] Guo Q., Wang X., Gao Y., Zhou J., Huang C., Zhang Z., Chu H. (2021). Relationship between particulate matter exposure and female breast cancer incidence and mortality: A systematic review and meta-analysis. Int. Arch. Occup. Environ. Health.

[9] Kelly F.J., Fussell J.C. (2020). Toxicity of airborne particles-established evidence, knowledge gaps and emerging areas of importance. Philos. Trans. A Math. Phys. Eng. Sci..

[10] Liang Q., Sun M., Wang F., Ma Y., Lin L., Li T., Duan J., Sun Z. (2020). Short-term PM(2.5) exposure and circulating von Willebrand factor level: A meta-analysis. Sci. Total Environ..

[11] Liang S., Zhang J., Ning R., Du Z., Liu J., Batibawa J.W., Duan J., Sun Z. (2020). The critical role of endothelial function in fine particulate matter-induced atherosclerosis. Part. Fibre Toxicol..

[12] Lin L., Li T., Sun M., Liang Q., Ma Y., Wang F., Duan J., Sun Z. (2021). Effect of particulate matter exposure on the prevalence of allergic rhinitis in children: A systematic review and meta-analysis. Chemosphere.

[13] Milici A., Talavera K. (2021). TRP Channels as Cellular Targets of Particulate Matter. Int. J. Mol. Sci..

[14] Ning J., Zhang Y., Hu H., Hu W., Li L., Pang Y., Ma S., Niu Y., Zhang R. (2021). Association between ambient particulate matter exposure and metabolic syndrome risk: A systematic review and meta-analysis. Sci. Total Environ..

[15] Sun M., Liang Q., Ma Y., Wang F., Lin L., Li T., Sun Z., Duan J. (2020). Particulate matter exposure and biomarkers associated with blood coagulation: A meta-analysis. Ecotoxicol. Environ. Saf..

[16] Wang F., Ahat X., Liang Q., Ma Y., Sun M., Lin L., Li T., Duan J., Sun Z. (2021). The relationship between exposure to PM2.5 and atrial fibrillation in older adults: A systematic review and meta-analysis. Sci. Total Environ..

[17] Zhu H., Wu Y., Kuang X., Liu H., Guo Z., Qian J., Wang D., Wang M., Chu H., Gong W. (2021). Effect of PM(2.5) exposure on circulating fibrinogen and IL-6 levels: A systematic review and meta-analysis. Chemosphere.

[18] Loxham M., Nieuwenhuijsen M.J. (2019). Health effects of particulate matter air pollution in underground railway systems—A critical review of the evidence. Part. Fibre Toxicol..

[19] Martins V., Moreno T., Minguillón M.C., Amato F., de Miguel E., Capdevila M., Querol X. (2015). Exposure to airborne particulate matter in the subway system. Sci. Total Environ..

[20] Smith J., Barratt B., Fuller G., Kelly F., Loxham M., Nicolosi E., Priestman M., Tremper A., Green D. (2019). PM2.5 on the London Underground. Environ. Int..

[21] Wen Y., Leng J., Shen X., Han G., Sun L., Yu F. (2020). Environmental and Health Effects of Ventilation in Subway Stations: A Literature Review. Int. J. Environ. Res. Public Health.

[22] Pétremand R., Wild P., Crézé C., Suarez G., Besançon S., Jouannique V., Debatisse A., Canu I.G. (2021). Application of the Bayesian spline method to analyze real-time measurements of ultrafine particle concentration in the Parisian subway. Environ. Int..

[23] Qiao T., Xiu G., Zheng Y., Yang J., Wang L., Yang J., Huang Z. (2015). Preliminary investigation of PM1, PM2.5, PM10 and its metal elemental composition in tunnels at a subway station in Shanghai, China. Transp. Res. D Transp. Environ..

[24] Loxham M., Cooper M.J., Gerlofs-Nijland M.E., Cassee F.R., Davies D., Palmer M.R., Teagle D.A.H. (2013). Physicochemical Characterization of Airborne Particulate Matter at a Mainline Underground Railway Station. Environ. Sci. Technol..

[25] Loxham M., Woo J., Singhania A., Smithers N.P., Yeomans A., Packham G., Crainic A.M., Cook R.B., Cassee F.R., Woelk C.H. (2020). Upregulation of epithelial metallothioneins by metal-rich ultrafine particulate matter from an underground railway. Met. Int. Biomet. Sci..

[26] Luglio D.G., Katsigeorgis M., Hess J., Kim R., Adragna J., Raja A., Gordon C., Fine J., Thurston G., Gordon T. (2021). PM2.5 Concentration and Composition in Subway Systems in the Northeastern United States. Environ. Health Persp..

[27] Van Ryswyk K., Kulka R., Marro L., Yang D., Toma E., Mehta L., McNeil-Taboika L., Evans G.J. (2021). Impacts of Subway System Modifications on Air Quality in Subway Platforms and Trains. Environ. Sci. Technol..

[28] Canu I.G., Hemmendinger M., Sauvain J.J., Suarez G., Hopf N.B., Pralong J.A., Ben Rayana T., Besançon S., Sakthithasan K., Jouannique V. (2021). Respiratory Disease Occupational Biomonitoring Collaborative Project (ROBoCoP): A longitudinal pilot study and implementation research in the Parisian transport company. J. Occup. Med. Toxicol..

[29] Canu I.G., Crézé C., Hemmendinger M., Ben Rayana T., Besançon S., Jouannique V., Debatisse A., Wild P., Sauvain J., Suárez G. (2021). Particle and metal exposure in Parisian subway: Relationship between exposure biomarkers in air, exhaled breath condensate, and urine. Int. J. Hyg. Environ. Health.

[30] Giordano M.R., Malings C., Pandis S.N., Presto A.A., McNeill V., Westervelt D.M., Beekmann M., Subramanian R. (2021). From low-cost sensors to high-quality data: A summary of challenges and best practices for effectively calibrating low-cost particulate matter mass sensors. J. Aerosol Sci..

[31] Masic A., Bibic D., Pikula B., Blazevic A., Huremovic J., Zero S. (2020). Evaluation of optical particulate matter sensors under realistic conditions of strong and mild urban pollution. Atmos. Meas. Tech..

[32] Kuula J., Mäkelä T., Aurela M., Teinilä K., Varjonen S., González Ó., Timonen H. (2020). Laboratory evaluation of particle-size selectivity of optical low-cost particulate matter sensors. Atmos. Meas. Tech..

[33] GRIMM Aerosol Technik (2010). Portable Laser Aerosolspectrometer and Dust Monitor Model 1.108/1.109. Users Manual.

[34] Cheng Y.-H., Lin Y.-L. (2010). Measurement of Particle Mass Concentrations and Size Distributions in an Underground Station. Aerosol Air Qual. Res..

[35] Reche C., Moreno T., Martins V., Minguillón M.C., Jones T., De Miguel E., Capdevila M., Centelles S., Querol X. (2017). Factors controlling particle number concentration and size at metro stations. Atmosph. Environ..

[36] van de Schoot R., Depaoli S., King R., Kramer B., Märtens K., Tadesse M.G., Vannucci M., Gelman A., Veen D., Willemsen J. (2021). Bayesian statistics and modelling. Nat. Rev. Methods Prim..

[37] Asbach C., Alexander C., Clavaguera S., Dahmann D., Dozol H., Faure B., Fierz M., Fontana L., Iavicoli I., Kaminski H. (2017). Review of measurement techniques and methods for assessing personal exposure to airborne nanomaterials in workplaces. Sci. Total Environ..

[38] Cheng Y.H. (2008). Comparison of the TSI Model 8520 and Grimm Series 1.108 portable aerosol instruments used to monitor particulate matter in an iron foundry. J. Occup. Environ. Hyg..

[39] Zuidema C., Stebounova L.V., Sousan S., Gray A., Stroh O., Thomas G., Peters T., Koehler K. (2020). Estimating personal exposures from a multi-hazard sensor network. J. Expo. Sci. Environ. Epidemiol..

[40] Zuidema C., Stebounova L.V., Sousan S., Thomas G., Koehler K., Peters T.M. (2019). Sources of error and variability in particulate matter sensor network measurements. J. Occup. Environ. Hyg..

[41] Burkart J., Steiner G., Reischl G., Moshammer H., Neuberger M., Hitzenberger R. (2010). Characterizing the performance of two optical particle counters (Grimm OPC1.108 and OPC1.109) under urban aerosol conditions. J. Aerosol Sci..

[42] Todea A.M., Beckmann S., Kaminski H., Bard D., Bau S., Clavaguera S., Dahmann D., Dozol H., Dziurowitz N., Elihn K. (2017). Inter-comparison of personal monitors for nanoparticles exposure at workplaces and in the environment. Sci. Total Environ..

[43] Mills J.B., Park J.H., Peters T.M. (2013). Comparison of the DiSCmini aerosol monitor to a handheld condensation particle counter and a scanning mobility particle sizer for submicrometer sodium chloride and metal aerosols. J Occup. Environ. Hyg..

