# Supplementary material for: A Real-Time Comparison of Four Particulate Matter Size Fractions in the Personal Breathing Zone of Paris Subway Workers: A Six-Week Prospective Study
Source: Sustainability. 2022 May 15;14(10):5999. doi: 10.3390/su14105999 (PMC9170000; doi:10.3390/su14105999)
Supplement: Supplementary file 1 [file sustainability-14-05999-s001.zip › sustainability-1712285-supplementary.pdf]

Supplementary Materials

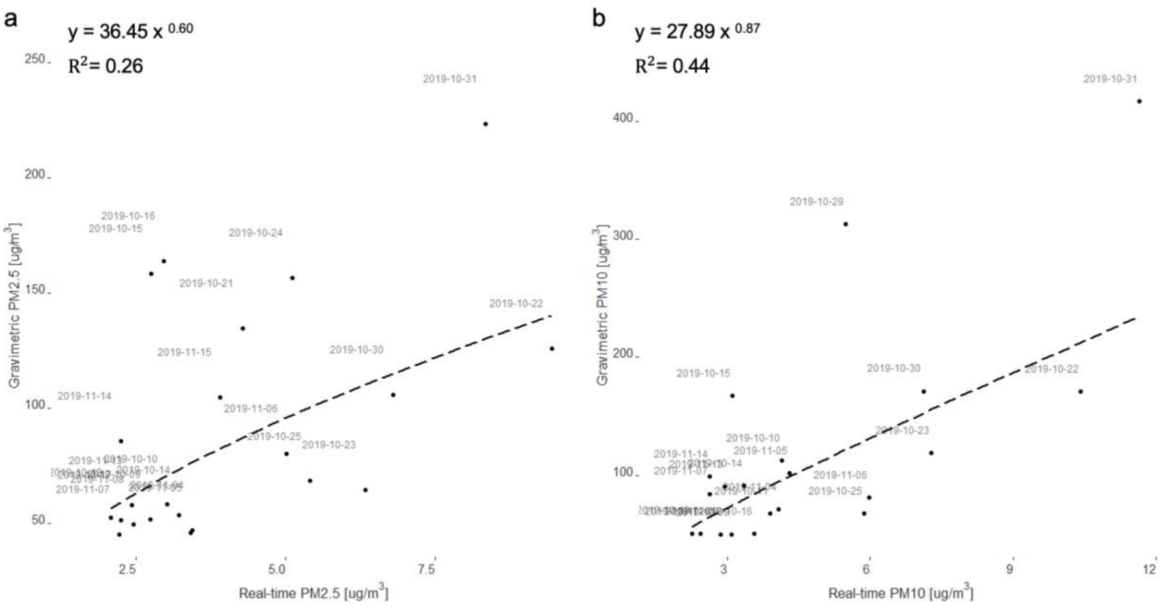

Figure S1: Regression analysis between the simultaneously collected gravimetric (Y-axis) and real-time GRIMM measurements (X-axis) of PM2.5 (a) and PM10 (b). The line represents the power curve that best fit the gravimetric and real-time PM data (with the highest R^2 fit). The recordings from the 28th October and 12th November 2019 were not taken into consideration as they were interrupted for several hours due to some technical limitations. Moreover, we also removed the data points from the 29th October 2019 for PM2.5 (a) and from the 21th October 2019 for PM10 (b) after they were identified as having a Cook's distance greater than 4/total sample number.

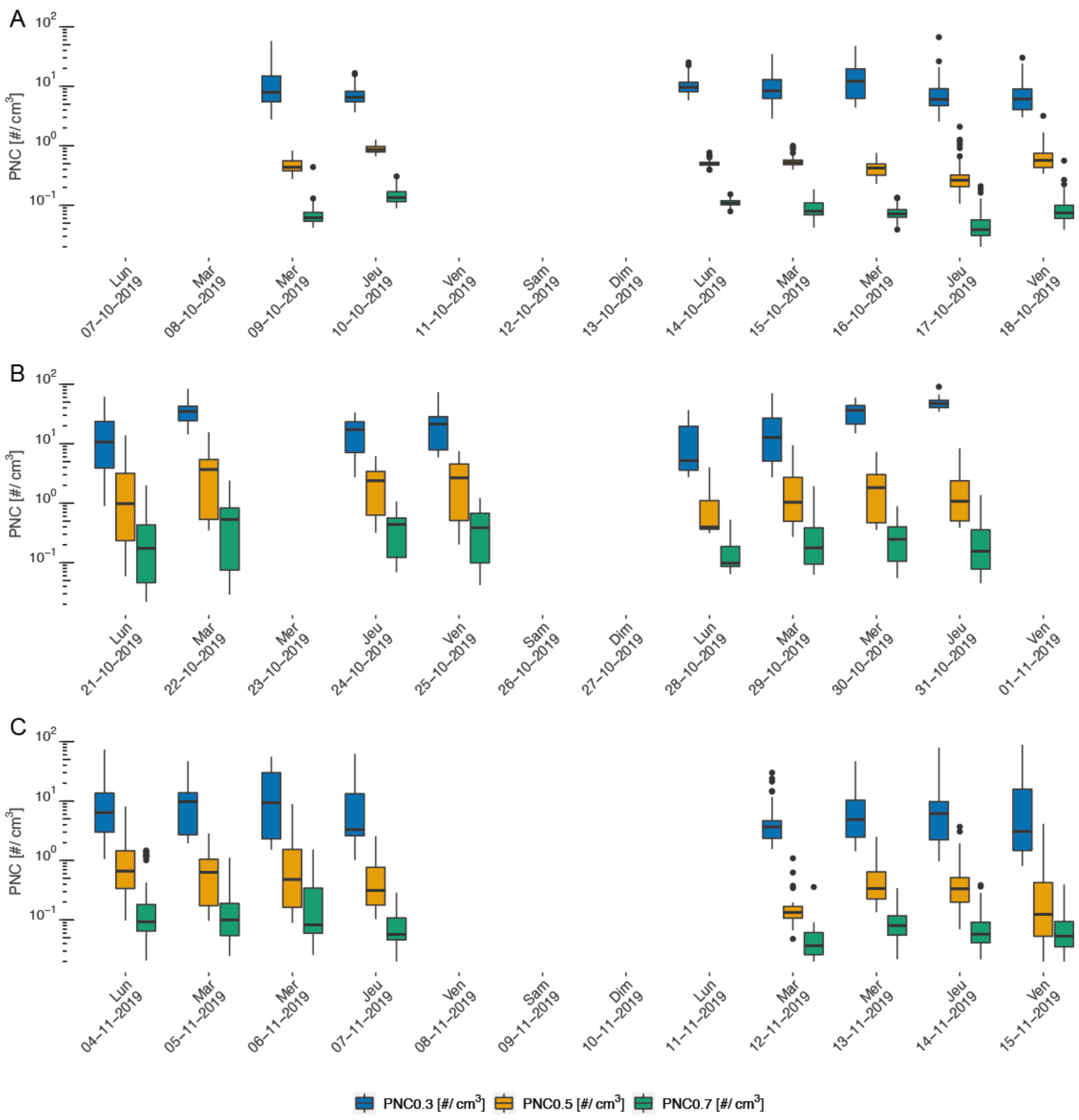

Figure S2: Particle number concentrations measured in the personal breathing zone samples of Parisian subway workers: stations agents (A), locomotive operators (B), and security guards (C).

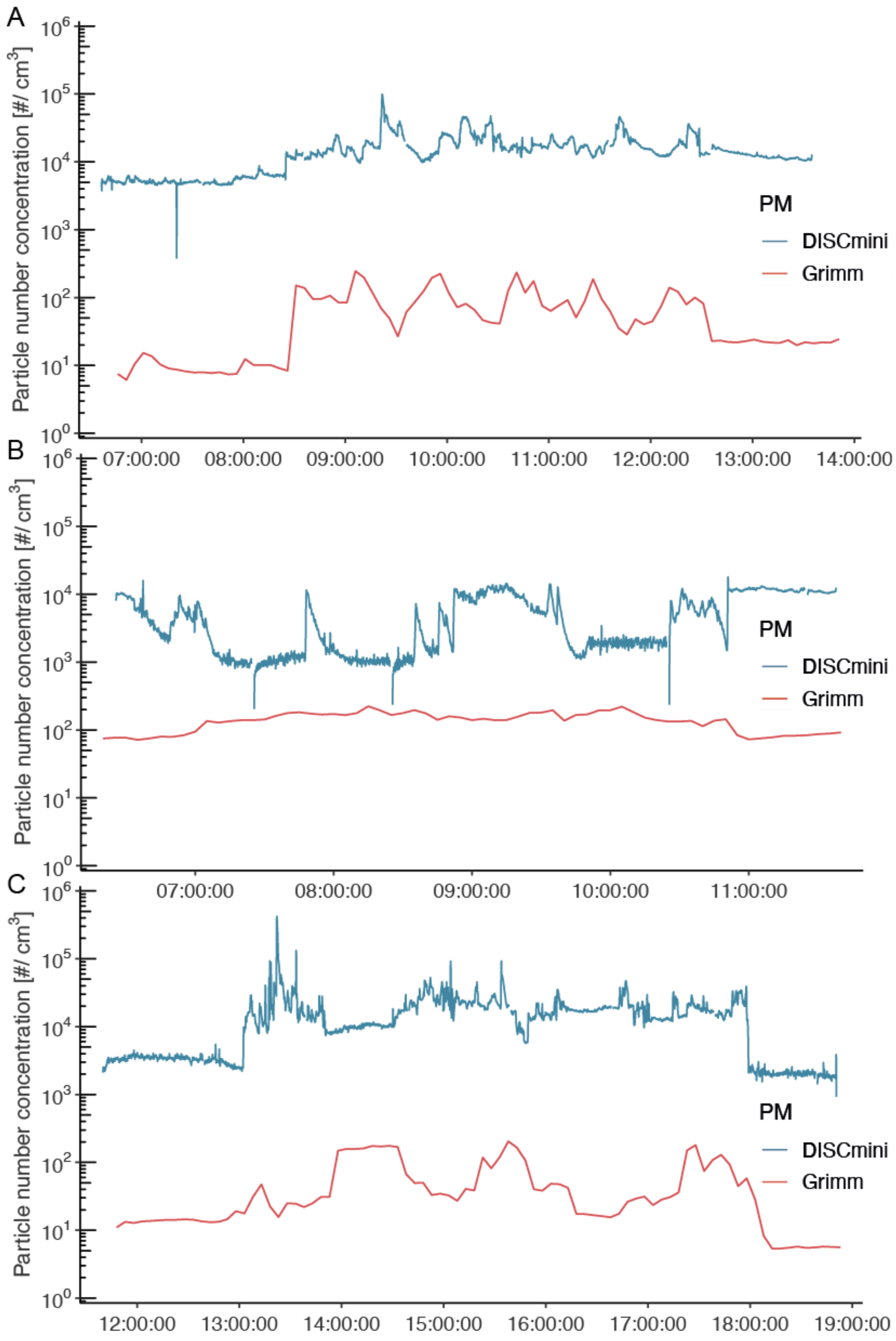

Figure S3: Integrated particle number concentration measured by DiSCmini for sizes from 0.01 to 0.7 µm (blue) and by GRIMM for sizes from 0.25 to 0.7 µm (red) in the personal

breathing zone samples of Parisian subway workers. (A) Station agents (21-10-2019), (B) locomotive operators (30-10-2019), and (C) security guards (4-11-2019).
